# Comparing the effects of hydrogen-infused and plasma-activated waters on the structure, physicochemical properties, and *in vitro* digestibility of mung bean starch under annealing treatment

**DOI:** 10.3389/fnut.2025.1665336

**Published:** 2025-09-03

**Authors:** Ge Tian, Shuang Liu, Shanshan Gao, Hang Liu

**Affiliations:** ^1^Shanxi Institute for Functional Food, Key Laboratory of Sustainable Dryland Agriculture of Shanxi Province, Shanxi Hou Ji Laboratory, Shanxi Agricultural University, Taiyuan, China; ^2^School of Food Science and Engineering, Hainan University, Haikou, China

**Keywords:** mung bean starch, annealing, plasma-activated water, hydrogen-infused water, physicochemical properties, digestibility

## Abstract

As far as we know, plasma-activated water (PAW) and hydrogen-infused water (HW) have been barely used in starch modification studies, especially bean starches. Therefore, the combined effect of annealing (ANN) and distilled water, HW, and PAW on the multi-structure, physicochemical properties, and *in vitro* digestibility of mung bean starch was investigated in this study. The scanning electron microscope results showed that the surface of annealed samples was much rougher than that of native mung bean starch (NMBS). Compared to that of NMBS, the typical “C”-type X-ray diffraction pattern of different annealed starches did not change, but their relative crystallinity increased. The annealed sample with PAW (PAnn) had the highest relative crystallinity (34.9%). Meanwhile, ANN significantly increased the amylose content, gelatinization properties, and water absorption capacity of mung bean starch, while it decreased swelling power and viscosity. The total hydrolysis rate and rapidly digestible starch content of mung bean starch significantly decreased after ANN, and the resistant starch level was markedly increased, with PAnn having the highest value (25.4%). The influence of ANN with HW and PAW on the above properties was much more extensive, respectively, than that of the annealed sample with distilled water, and the PAnn had the highest value, which might be based on the acidic environment and reactive oxygen and nitrogen species. These results from this research not only provided a novel strategy for improving the thermal stability and functionality of mung bean starch but also extended the application of HW and PAW in starch modification.

## 1 Introduction

Mung bean (*Vigna radiata* L.) is an edible crop belonging to the legume family. It originated in China and has been cultivated for more than 3,500 years (1), which is mainly distributed in Southeast Asia, Africa, South and North America, and Australia (2). In recent decades, various studies have shown that mung bean is a rich source of nutrients, including phenolic compounds, minerals, vitamins, lipids, protein, and starch (3). These properties have endowed mung bean with antidotal, antibacterial, antioxidant, antidiabetic, anti-hyperlipidemia, and anti-tumor capacities (4). Traditionally, mung beans have been applied into porridge, snacks, beverages, etc. as whole grains and into cakes or sweetened bean pastes after grinding and milling. Meanwhile, sprouts germinated from mung beans are eaten as a popular vegetable in China since they are a good and inexpensive source of high nutrition (5). However, as a “hard-to-cook” legume, consumption of mung beans is tricky, which has limited its application in the food industry (6). Starch is the dominant component in mung bean grain, accounting for ~50–60% of dry weight, which contains an amylose content in the range from 30% to 45% based on variety and growing environment (7). Although mung bean starch has been considered as a new starch resource and applied in soup, sauces, and bakeries as a thickener, the drawbacks of high syneresis, poor storage stability, and low swelling power of native mung bean starch (NMBS) greatly restrict its applications (8). Therefore, to meet the demand for specific purposes, modification of mung bean starch is urgent to improve its performance for further wide application in the food industry.

Generally, various methods have been used for starch modification, including chemical, physical, enzymatic, and a combination of these methods (7). Since physical modification of starch is not only environmentally friendly but also easily controlled, it has been widely studied and popularly used in the recent decade. Several physical modification methods have been applied to study the properties of mung bean starch, such as heat-moisture treatment (9), high hydrostatic pressure (10), microwave treatment (11), dry heating (7), etc. Among these methods, annealing (ANN) has gained long-standing concern based on its low cost, simple, and practical process (12). As “clean-labeled” technology, ANN is carried on with excess water at a temperature between the glass transition and the gelatinization temperature of native starch. Previous studies have indicated that ANN would increase relative crystallinity (RC), raise water absorption capacity, decrease viscosity, and improve resistant starch level without destroying granules (13). These changes in annealed starches improved their application in manufacturing noodles, bread, and canned foods. However, the systematic influence of ANN on the properties of mung bean starch with different types of water has not been studied.

In recent years, plasma-activated water (PAW) has been produced by treating distilled water with plasma. Because it contains highly reactive oxygen and nitrogen species (RONS) like H_2_O_2_, ${\text{NO}}_{2}^{-}$, ${\text{NO}}_{3}^{-}$, and O_3_, PAW has a low pH, high oxidation–reduction activity, and electrical conductivity (14). Although PAW has been broadly utilized in food and agriculture areas for preserving seafood, promoting plant growth, and sterilizing products (15), it has barely been applied in research on starch modification. As an antioxidant, molecular hydrogen (H_2_) has been proven to have therapeutic value in the human body by selectively reducing cytotoxic reactive oxygen species (ROS) (16). Meanwhile, hydrogen inhalation (2%) could also significantly ameliorate the inflammation during intestinal transplant injury through its antioxidant effects (17). Recently, drinking hydrogen-infused water (HW) with a therapeutic dose of hydrogen has been an alternative choice for delivering molecular hydrogen into humans, which is a portable, easily administered, and safe way. However, HW is rarely applied to starch modification either, and its effects on starch properties are not clear. Furthermore, according to these preliminary studies, the use of PAW alone had a negligible influence on the structure and properties of starches. Therefore, combining PAW or HW with physical technologies can be an innovative strategy for starch modification. In this study, both PAW and HW were used along with ANN to alter the structure, physicochemical properties, and *in vitro* digestibility of mung bean starch. These results will provide a new way to modify mung bean starch and further enrich the evidence for PAW and HW to expand their application into the starch modification area.

## 2 Materials and methods

### 2.1 Materials

Grains of the new mung bean variety (BL01-55) were provided by the Center for Agricultural Genetic Resources Research, Shanxi Agricultural University. The HW was provided by Shanxi Yuemingtang Biotechnology Co., Ltd., Taiyuan, China. Enzymes, including pepsin (P7125; ≥400 U/mg), porcine pancreatic α-amylase (A3176; 5 U/mg), and amyloglucosidase from *Aspergillus niger* (A9913), were purchased from Sigma-Aldrich (Shanghai) Trading Co. Ltd. (Shanghai, China). The glucose oxidase-peroxidase (GOPOD) assay kit was purchased from Megazyme International Ireland Ltd. (Bray, Ireland). All other chemicals were of analytical grade.

### 2.2 Starch isolation

The mung bean starch was isolated using a wet milling scheme published by Liu et al. (18) with minor modifications. The initial deposition was washed with water and centrifuged at 975 × *g* for 10 min to remove impurities. Then, 0.2% sodium hydroxide solution was added to eliminate the protein. After centrifuging, the protein layer was manually scraped, and the precipitate was washed with distilled water at least three times until the supernatant was clear and no longer yellow. Afterward, the obtained NMBS was dried at 40 °C for 24 h in a constant temperature convection oven and ground into powder for further research.

### 2.3 Preparation and characterization of PAW

The PAW was obtained by treating distilled water with a jet plasma device (PG-1000ZD, Nanjing Suman Plasma Engineering Research Institute Co., Ltd, China) under atmospheric pressure at 750 W for 120 s, which must be used within 12 h. The pH, oxidation–reduction potential (ORP), and electrical conductivity of the waters were, respectively, tested by a pH/ORP meter and an electric conductivity meter (Yidian Scientific Instrument Co. Ltd., Shanghai, China). The content of H_2_O_2_, ${\text{NO}}_{2}^{-}$, and ${\text{NO}}_{3}^{-}$ in the different waters was determined using corresponding assay kits.

### 2.4 Annealing treatment

The ANN treatment was conducted using the method from Liu et al. (12). The starch slurry was obtained by dispersing NMBS into distilled water, HW, and PAW at a ratio of 1:4 (w/v), which were sealed in containers to equilibrate overnight at 4 °C. Subsequently, the containers were annealed for 24 h at 50 °C in a constant temperature convection oven. The samples were air-dried at 40 °C after cooling, and the annealed samples were referred to as DAnn, Hann, and PAnn based on the type of water used.

### 2.5 Scanning electron microscope (SEM) observation

The morphology of samples was observed using a scanning electron microscope (SEM; Regulus8240, Hitachi Ltd., Tokyo, Japan). Samples were mounted on a double-sided adhesive tape attached to a metal stub, which was coated with 20-nm gold layer under vacuum. Then, the samples were imaged under an acceleration potential of 20 kV.

### 2.6 X-ray diffraction (XRD)

The XRD pattern and relative crystallinity (RC, %) of starches were tested with an X-ray diffractometer (D/MAX 2500 V, Rigaku Corporation, Japan). Under 40 kV and 30 mA current, the samples were scanned from 5° to 60° (2θ) at a rate of 4 °/min. The RC values were calculated using Jade 6.0 software (OriginLab Corporation, USA).

### 2.7 Fourier transform-infrared (FT-IR) spectroscopy

The FT-IR spectra of the samples were obtained using a Fourier transform-infrared spectrometer (Vertex 70, Bruker, Karlsruhe, Germany) at wave numbers from 4,000 to 400 cm^−1^.

### 2.8 Water and oil absorption capacities

The water and oil absorption capacities of different starches were determined by a method reported by Liu et al. (19). The starch samples (4 g, [dry weight, db]) and 20 mL water or peanut oil were transferred to 50-mL centrifuge tubes, stirred with a glass rod for 30 min at 30 °C, and centrifuged at 7,385 × *g* for 15 min. The volume of the decanted supernatant was measured, and the volume of water or oil retained per gram of sample was calculated (in mL/g).

### 2.9 Alkaline water retention and amylose content

Alkaline water retention (AWR) of different starches was measured using the method published by Adebowale et al. (20). Sample (1.0 g, db) was transferred into a tube and weighed (W_1_). Subsequently, 0.1 M NaHCO_3_ (5 mL) was added and mixed for 30 s. The mixture was allowed to stand for 20 min at 30 ± 2 °C. After incubation, the tube was centrifuged (35 × *g*, 15 min) and drained for 10 min at an angle of 10–15° with respect to the horizontal. The tube with contents was weighed (W_2_) again. The AWR was calculated as follows,


(1) $$\begin{array}{l} \text{AWR (g/g) of sample}=\;{\text{W}}_{\text{2}}-{\text{W}}_{\text{1}} \end{array}$$

The procedure reported by Juliano et al. (21) was conducted to determine the amylose content (AMC) of all samples. In brief, the defatted starch (100 mg, db) and 1 M NaOH were equilibrated in a flask for 24 h at room temperature. The solution volume was made up to 100 mL with distilled water and vigorously mixed. Starch dispersion (5 mL), 1 M acetic acid, and 2 mL of iodine solution were mixed and made up to 100 mL again with distilled water. After 20 min, the absorbance of the mixture was determined at 620 nm.

### 2.10 Solubility and swelling power (SP)

The SP and solubility of different starches were tested according to the method from Liu et al. (22). Sample (50 mg, db) was transferred into dry centrifugal tubes, weighed, and mixed with distilled water (5 mL). The tubes were incubated in a shaking water bath at 50, 60, 70, 80, and 90 °C for 30 min, cooled to room temperature, and centrifuged at 657 × *g* for 15 min. The supernatant was carefully decanted, and the resulting tubes with their contents were weighed. The residue obtained after drying the supernatant represented the amount of starch dissolved in water. Solubility and SP were calculated on a dry weight basis using the following equations:


(2) $$\begin{array}{lll} \text{Solubility} & = & \frac{\text{the weight of dried supernatant}}{\text{weight of starch}} \end{array}$$

(3) $$\begin{array}{lll} \text{SP} & = & \frac{{\text{W}}_{2}-{\text{W}}_{1}}{\text{weight of starch}} \end{array}$$

where W_1_ represents the weight of the tube with the starch sample, and W_2_ represents the weight of the tube with the resulting precipitate after decanting the supernatant.

### 2.11 Differential scanning calorimetry (DSC)

Gelatinization characteristics of starches were assessed with a differential scanning calorimeter (Q2000, TA Instruments, New Castle, DE, USA). Starch (3 mg, db) was sealed in a sample pan after mixing with 9 mL DW and equilibrated overnight. With an empty sample pan as control, the samples were heated from 30 to 130 °C at a rate of 10 °C/min. The onset temperature (*T*_o_), peak temperature (*T*_p_), conclusion temperature (*T*_c_), and gelatinization enthalpy (Δ*H*) were determined.

### 2.12 Pasting properties

The pasting properties of different samples were tested with a Rapid Visco Analyzer (RVA-4, Newport Scientific Co., Ltd., Warriewood, NSW, Australia) based on the procedure reported by Liu et al. (22). The setback viscosity (SB), breakdown viscosity (BD), peak viscosity (FV), pasting temperature (PT), and final viscosity (FV) were measured.

### 2.13 *In vitro* digestibility

#### 2.13.1 *In vitro* digestion of starch

*In vitro* digestibility of different starches was determined according to a published research of Liu et al. (22). Starch (50 mg, db) was put into a 50-mL flask with 5 mL of distilled water. After gelatinization and cooling, 10 mL of HCl-KCl buffer (0.05 M, pH l.5) and 0.2 mL of the pepsin solution were added to each flask. It was incubated in a shaking water bath at 40 °C for 60 min. Subsequently, the total sample volume was adjusted to 25 mL with sodium acetate buffer (0.5 M, pH 6.9). Amylase solution (5 mL, 2.6 UI) was added and incubated in a shaking water bath at 37 °C for 3 h. Aliquots (1 mL) were obtained from each flask every 10 min during the first 30 min of hydrolysis and every 30 min during the remaining 2.5 h of hydrolysis. The obtained samples were transferred into dry centrifuge tubes, which were immediately placed in boiling water to inactivate the amylase. After cooling, sodium acetate buffer (0.4 M, pH 4.75, 3 mL) and amyloglucosidase (60 μL) were added to each tube. The tubes were then shaken at 60 °C for 45 min. The GOPOD kit was used to determine the glucose content of samples, and the starch amount was calculated by multiplying the glucose content by 0.9. The digestion rate was expressed as the percentage of total starch hydrolyzed after different periods.

#### 2.13.2 Resistant starch (RS) content

The levels of rapid digestible starch (RDS), slow digestible starch (SDS), and resistant starch (RS) were calculated according to the hydrolysis curve. RDS is the starch digested in the first 20 min, while SDS is the starch digested between 20 and 120 min. The RS is the starch remaining after 180 min.

### 2.14 Statistical analysis

All data were triply measured to gain mean values and standard deviations, which were analyzed by one-way analysis of variance (ANOVA) using SPSS software (version 20.0, SPSS Inc., Chicago, IL, USA). The statistical significance was set at a level of *p* < 0.05. Principal component analysis (PCA) was performed using Minitab version 17 (Minitab Inc., USA).

## 3 Results and discussion

### 3.1 Properties of the waters

The properties of three different waters, especially the content of some active substances, are shown in Table 1. The pH of HW was 8.3, containing more than 3.0 μmol/L hydrogen atoms, both of which were significantly higher than those of distilled water. The chemical reactions that occurred between the interface of plasma and liquid would generate active substances, including singlet oxygen, ozone, hydroxyl radicals, and active molecule nitrogen species, to change the pH, ORP, and electric conductivity values of the liquid (23). In this study, the pH of distilled water significantly decreased from 6.70 to 2.32 after being treated with plasma. This demonstrates that plasma treatment can acidify water. Compared with those of distilled water, the electric conductivity of PAW markedly increased from 17.97 to 1,123.34 μS/cm, and the ORP also increased from 266.30 to 577.36 mV, probably by the generation of ROS (24). This improved conductivity of PAW might be due to the development of soluble active groups and ions (ROS or RNS) (25), which further resulted in oxidation–reduction performance in PAW. In addition, the content of H_2_O_2_, ${\text{NO}}_{2}^{-}$, and ${\text{NO}}_{3}^{-}$ increased from 0 to 158.25, 1,987.12, and 2,172.36 μmol/L, respectively. They were consistent with the above results.

**Table 1:** Properties and content of active substances in different waters.

**Types of water**	**pH**	**ORP (mV)**	**Conductivity (μS/cm)**	**${\text{NO}}_{3}^{-}$(μmol/L)**	**${\text{NO}}_{2}^{-}$(μmol/L)**	**H_2_O_2_ (μmol/L)**	**Hydrogen atom (μmol/L)**
DW	6.70 ± 0.55^b^	266.30 ± 0.19^c^	17.97 ± 1.15^c^	-	-	-	-
HW	8.30 ± 0.45^a^	356.52 ± 0.27^b^	268.25 ± 0.68^b^	-	-	-	> 3.0
PAW	2.32 ± 0.73^c^	577.36 ± 0.79^a^	1,123.34 ± 1.05^a^	2,172.36 ± 0.51	1,987.12 ± 1.75	158.25 ± 0.45	-

Means of triplicate determination ± SD with different letters in a column within each property are significantly different (*p* < 0.05). DW: distilled water; HW: hydrogen water; PAW: plasma-activated water; ORP: oxidation–reduction potential; “-”: not detected.

### 3.2 Morphology of starches

**Figure 1:**
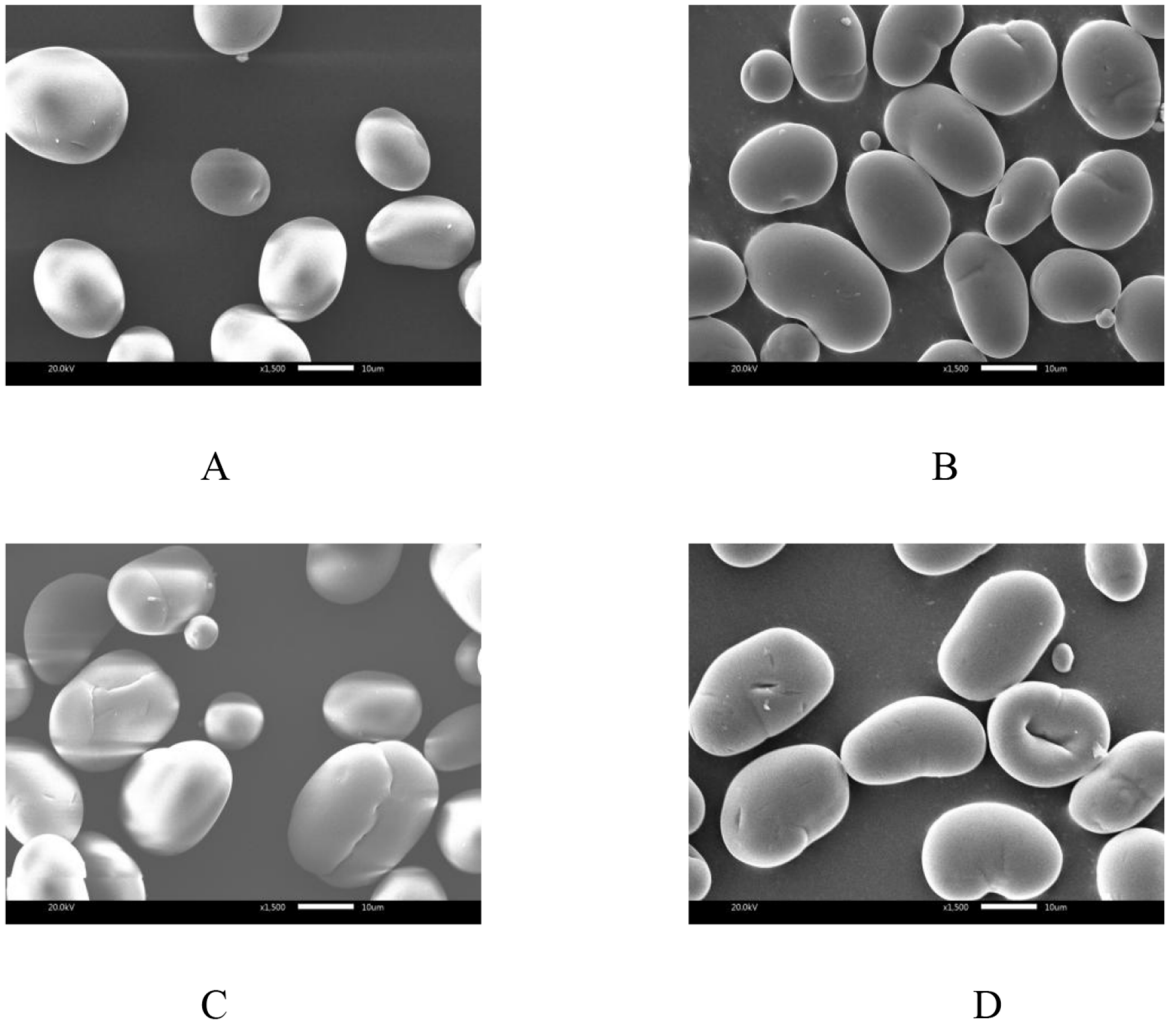
The SEM photos (1.5 k ×) of different samples. **(A)** NMBS; **(B)** DAnn; **(C)** HAnn; **(D)** PAnn.

The SEM images of native and annealed starches are shown in Figure 1. The surface of NMBS granules was smooth, with a mixture of kidney, spherical, and ellipse shapes varying with size (Figure 1A). After the ANN treatment, some pits were observed on the surface of DAnn samples (Figure 1B). Compared to DAnn, HAnn granules had fissures and even some cavities on their surfaces (Figure 1C), while many deeper dents and holes were found on PAnn granules (Figure 1D). The degree of morphology change in the different samples was significantly related to the difference among the three types of water. Similar results have also been reported on talipot starch annealed with PAW (26).

Generally, ANN can induce the recombination of amylose and amylopectin molecules in starch granules, which contributes to a more compact amorphous structure and leads to the morphology alteration of the starch granule (12). This is the main mechanism for the morphological changes in DAnn. The morphological changes in HAnn were worse than those in DAnn, which might be ascribed to redox reactions that happened on the granule surface, resulting from hydrogen atoms and hydrogen molecules in HW. Furthermore, the various active substances and acidic environment of PAW contributed to more transformation or rearrangement of central molecules in NMBS granules in this study. This would not only induce severe surface degradation of PAnn but may also facilitate its hydrolysis rate. These results indicate that the degree of starch morphological changes was dependent on the type and properties of water used during ANN.

### 3.3 XRD analysis

**Figure 2:**
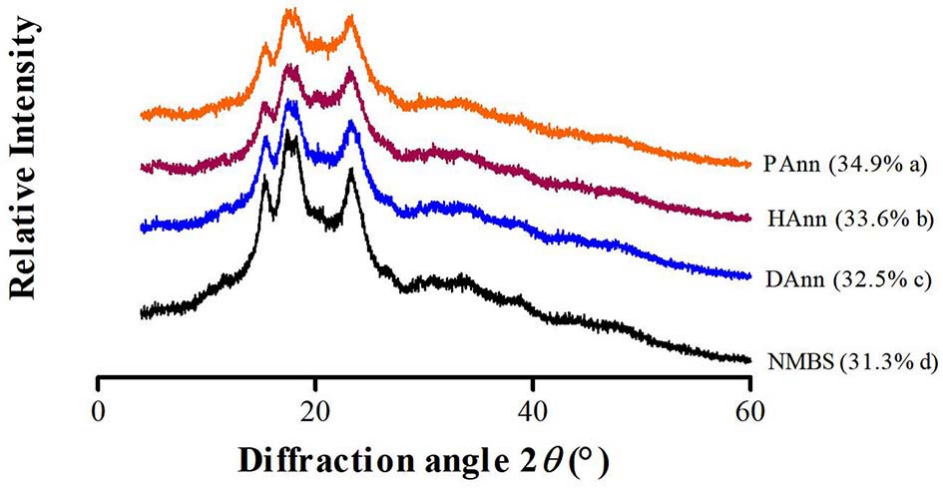
XRD pattern and RC (in parenthesis) of different starches.

XRD patterns of native and annealed samples with RC are shown in Figure 2. The NMBS had four characteristic diffraction peaks at 2θ angles of 15.33, 17.41, 17.91, and 23.37°, which indicated a typical “C”-type crystalline pattern. In comparison with NMBS, all annealed samples had diffraction peaks at similar diffraction angles. This indicates that the XRD pattern of NMBS was not altered by ANN with the three types of water. According to a previous report by Yan et al., the crystalline pattern of potato and pea starches was not affected under ANN with PAW (27).

The RC of NMBS was 31.3%, which had been significantly increased after ANN in the following order: DAnn (32.5%) < HAnn (33.6%) < PAnn (34.9%). A similar increase in RC had also been found on buckwheat and sorghum starches following ANN as well (12). This RC increment in annealed starches was related to various factors during ANN, like increased AMC, increased crystalline size, improved new crystallite formation by interactions between starch chains, and enhanced crystalline perfection (28). More aggregation of small crystallites and organization of double helix chains in NMBS granules might be other reasons for the increase in the RC (29). In addition, the RC values of HAnn (33.6%) and PAnn (34.9%) were significantly higher (*p* < 0.05) than that of DAnn (32.5%). This increase by the participation of HW might be due to the preferential hydrolysis of amorphous regions in starch caused by the alkaline environment, while the acidic environment and RONS present in PAW were the main factors for the RC increase in PAnn. These would improve parallel packing of double helices or reorientation of unpacked double helices within the crystalline array of starch granules.

### 3.4 FT-IR spectroscopy analysis

**Figure 3:**
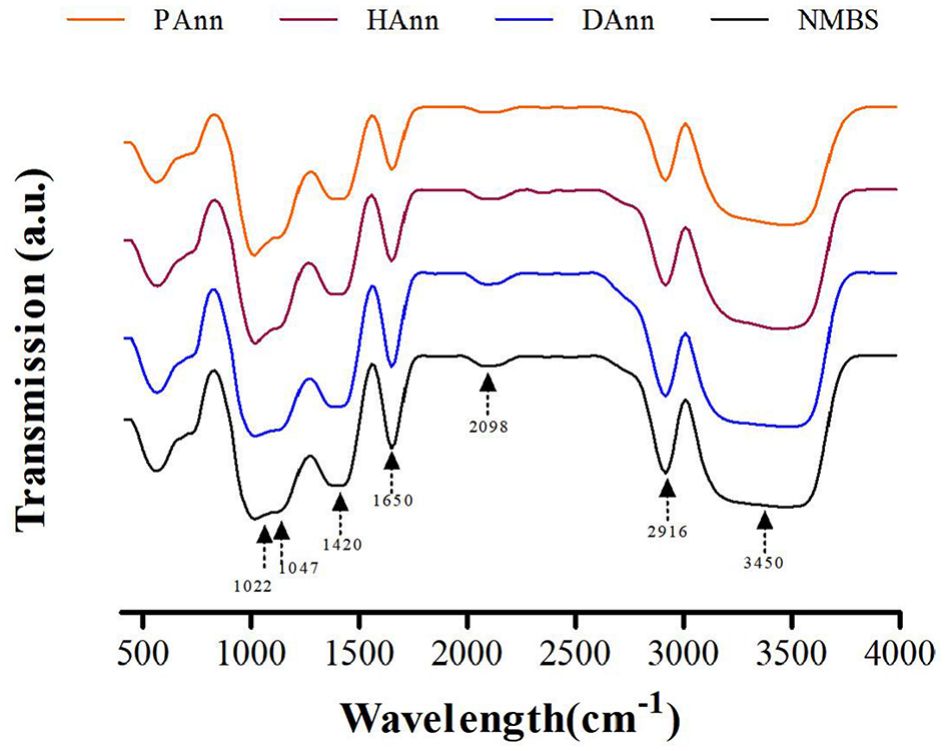
FT-IR spectra of all starch samples.

The FT-IR spectra of different starches were scanned at wavelengths from 400 to 4,000 cm^−1^ (Figure 3), and the values of R_1047/1022_ are shown in Table 2. Compared to NMBS, the annealed starches had similar absorption peaks on the spectra to show neither new functional groups formed nor existing groups were destroyed during ANN under the current circumstances. In general, the FT-IR band recorded at 1,022 cm^−1^ represents amorphous content in granules, while the band recorded at 1,047 cm^−1^ is related to an ordered crystalline structure (30). Therefore, the ratio of 1,022 and 1,047 cm^−1^ absorbance (*R*_1047/1022_) can indicate the relative content of short-range ordered structure domains in starch (27). The *R*_1047/1022_ of native and annealed starches followed the order: NMBS < DAnn < HAnn < PAnn. The *R*_1047/1022_ ratios of HAnn and PAnn were significantly higher than those of DAnn and NMBS. This increase in *R*_1047/1022_ of DAnn mainly depended on superfluous water and moderate heating conditions of ANN, which improved the double helix stacking in starch granules (13). Meanwhile, the increase in *R*_1047/1022_ of HAnn might be correlated with the cross-link between starch chains facilitated by hydrogen ions, hydrogen atoms, and the high pH of HW. The increase in *R*_1047/1022_ was attributed to the formation of new double-helical structures from short-chain amylose during ANN generated by RONS and acidic environment in PAW (27). These findings were in agreement with the results of XRD.

**Table 2:** AMC, AWR, WAC, OAC, and *R*_1047/1022_ of different samples.

**Parameters**	**Samples**			
	**NMBS**	**DAnn**	**HAnn**	**PAnn**
AMC (%)	29.40 ± 0.35^d^	30.10 ± 0.23^c^	31.62 ± 0.11^b^	33.75 ± 0.15^a^
AWR (g/g)	1.47 ± 0.21^c^	1.53 ± 0.34^c^	1.83 ± 0.29^b^	1.96 ± 0.25^a^
WAC (g/g)	1.68 ± 0.28^d^	1.85 ± 0.35^c^	2.21 ± 0.31^b^	2.45 ± 0.19^a^
OAC (g/g)	1.56 ± 0.34^a^	1.47 ± 0.28^b^	1.35 ± 0.16^c^	1.21 ± 0.35^d^
*R*_1047/1022_	0.93 ± 0.11^c^	0.94 ± 0.25^c^	0.98 ± 0.22^b^	1.04 ± 0.16^a^

Means of triplicate determination ± SD with different letters in a row within each property are significantly different (*p* < 0.05). AWR: alkaline water retention; AMC: amylose content; WAC: water absorption capacity; OAC: oil absorption capacity; R_1047/1022_: the ratio of absorbance at 1047/1022 cm^−1^ in FT-IR.

### 3.5 Water and oil absorption capacities

As shown in Table 2, there are significant differences in water and oil absorption capacities (WAC and OAC) among NMBS and annealed starches. Compared to NMBS, annealed starches had higher WAC, but poorer OAC. The PAnn had the highest (2.45 ± 0.19 g/g) and lowest (1.21 ± 0.35 g/g) values, respectively. These results indicate a stronger interaction between hydroxyl and water molecules within the starch granules during ANN. This interaction led to opposing tendency changes in the hydrophilicity and hydrophobicity of NMBS.

A few hydrogen bonds between amorphous and crystalline regions were destroyed during ANN to improve the expansion of the amorphous region. This significantly increased the hydrophilic tendency of starch molecules, further generating higher WAC in annealed starches (20). A reorganization of double helices induced by HW and PAW would stimulate the NMBS to hold more water (27), which mainly depended on the structure of flexible amylopectin. It was an important factor for the higher WAC of HAnn and PAnn than that of DAnn. This increased WAC had also been found in annealed tartary buckwheat and sorghum starches (12), and applying annealed starches into dough improved the color, volume, and textural properties of bread (31). All above-identified annealed starches could be used to promote the quality of baked products, especially HAnn and PAnn. However, the underlying principles of ANN affecting the WAC and OAC of starches are still limited. Therefore, further research is needed to fully explain this phenomenon.

### 3.6 AWR and AMC results

The AWR and AMC of different starches are shown in Table 2. The AWR is an important parameter of starch affecting its processing and application, which can represent the spread potential of dough, particularly can be used to predict the cookie diameter during baking (32). Following ANN, a significant increase in AWR of NMBS had been found. Similar results had also been reported on annealed sorghum and buckwheat starches (12). These increases were mainly attributed to starch surface area and excessive dilution at high starch concentrations during ANN (33). Furthermore, compared to that of NMBS, the higher WAC of annealed starches might also be responsible for increasing AWR. These results indicate that annealed starches are more suitable for making cookies than NMBS, with PAnn being much better than DAnn and HAnn.

Compared with NMBS, the annealed starches had remarkably higher AMC: PAnn had the highest increase (4.35%), while DAnn had the lowest (0.70%). During ANN, the degradation of amylopectin was the main reason for the increase in AMC (28). Meanwhile, ANN influenced the mobility of both amorphous and crystalline regions of NMBS. These changes resulted in reorganization and interaction of amylose-amylose, amylose-amylopectin, or amylopectin-amylopectin, which would improve the iodine-binding capacity (34). Furthermore, the limited amylose leaching might be another factor to increase AMC in annealed samples. The PAW had more extensive reactive species than distilled water, while the HW had significantly higher hydrogen atoms. These would induce more networks and cross-links in starch granules during ANN to further facilitate iodine-amylose complex formation. Gao et al. (35) reported similar results on tartary buckwheat starch modified by direct dielectric barrier plasma.

### 3.7 Solubility and swelling power (SP)

**Figure 4:**
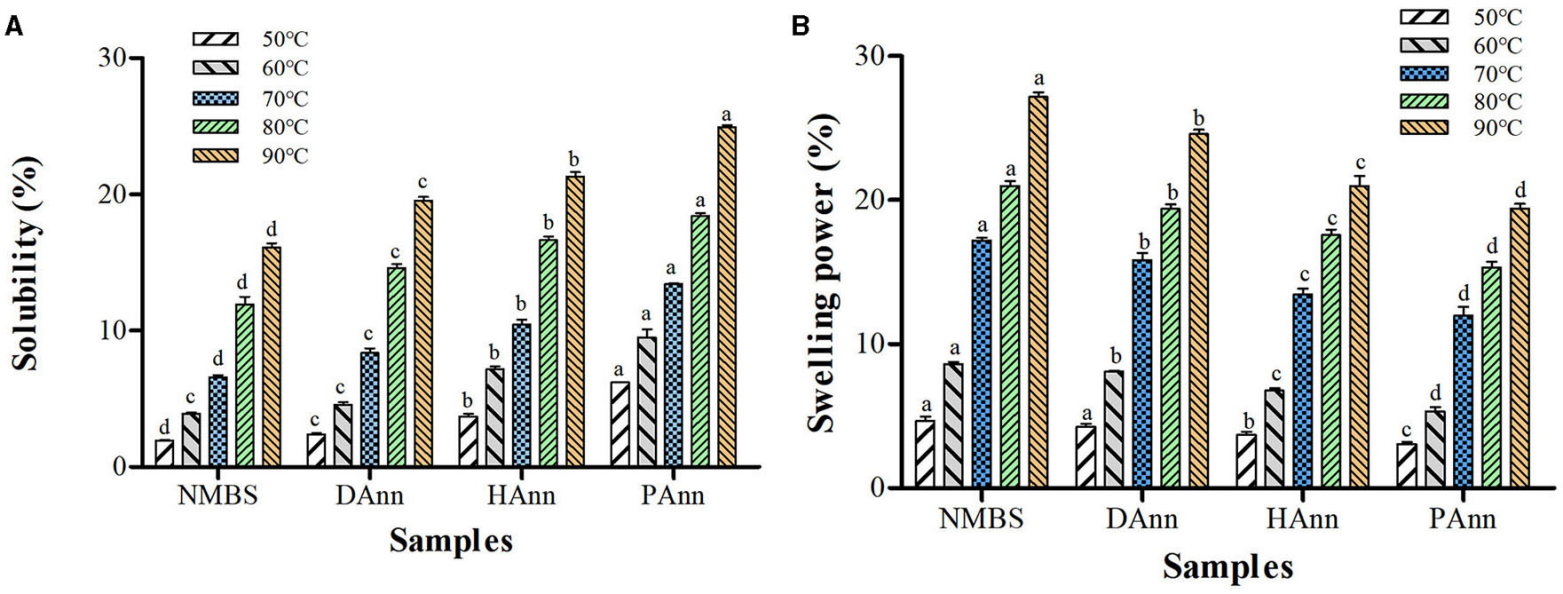
Solubility **(A)** and SP **(B)** of native and annealed samples. Bars bearing the same letter within same temperature are not significantly different (*p* < 0.05).

The influence of test temperature on solubility and SP of native and annealed starches is shown in Figures 4A, B, respectively. The solubility and SP of each sample increased with test temperature (50–90 °C). However, opposite alteration trends had been found in these two properties after ANN. Generally, the solubility of starch is a consequence of amylose leaching. Thus, in this study, the increase in solubility at each temperature mainly depended on the destruction of the double-helical structure in amylopectin during ANN, which increased the leaching of amylopectin by thermal damage and improved WAC as well (36). The alkaline environment in HAnn and the acidic substances and RONS in PAnn made the starch easier to separate and diffuse than DAnn, generating higher solubility (27). Compared to that of NMBS, the SP of annealed samples significantly decreased at each temperature, mainly based on the decrease in water retention ability of amylopectin. Meanwhile, the rearrangement among starch chains and improvement of crystalline perfection during ANN restricted starch hydration, which remarkably reduced the SP of annealed starches (37). Furthermore, changes in annealed starch granules, including increased AMC, improved small networks, and stronger molecular interactions, also contributed to the SP reduction (38). As shown in Figure 4, the solubility of HAnn and PAnn gradually increased than that of DAnn, while the SP significantly decreased. These were due to the decrease in water-binding ability of starch molecules that resulted from alkaline or acid hydrolysis of the amorphous region, respectively.

### 3.8 DSC analysis

The gelatinization temperatures (*T*_o_, *T*_p_, and *T*_c_) and enthalpy (Δ*H*) of different starches are shown in Table 3. The gelatinization temperatures and Δ*H* of annealed samples were significantly increased compared to those of NMBS, and the PAnn had the highest values. Similar results had been found on pea, maize, and non-conventional talipot starches (26, 27, 39), while inconsistent results of Δ*H* had also been reported for potato starch following ANN (27). These findings suggest that the combined effect of ANN and different types of water on Δ*H* is dependent on the starch source.

**Table 3:** Gelatinization characteristics of native and annealed samples.

**Samples**	***T*_o_ (°C)**	***T*_p_ (°C)**	***T*_c_ (°C)**	***T*_c_ – *T*_o_ (°C)**	**Δ*H* (J/g)**
NMBS	65.2 ± 0.41^d^	70.6 ± 0.32^d^	78.7± 0.17^c^	13.5 ± 0.25^a^	7.7 ± 0.18^c^
DAnn	66.9 ± 0.18^c^	71.1 ± 0.22^c^	79.2 ± 1.01^c^	12.3 ± 0.31^b^	8.3 ± 0.15^c^
HAnn	69.7 ± 0.21^b^	74.2 ± 0.27^b^	80.6 ± 0.42^b^	10.9 ± 0.26^c^	9.1 ± 0.35^b^
PAnn	72.8 ± 0.47^a^	77.3 ± 0.16^a^	82.5 ± 0.23^a^	9.7 ± 0.21^d^	10.8 ± 0.24^a^

Means of triplicate determination ± SD with the same letter in a column within each parameter are not significantly different (*p* < 0.05). *T*_o_: onset temperature; *T*_p_: peak temperature; *T*_c_: concluding temperature; *T*_c_-*T*_o_: gelatinization temperature range; Δ*H*: transition enthalpy.

The interactions between amylose and amylopectin or amylose during ANN would limit starch granules' swelling to delay their gelatinization, further increasing the temperatures of annealed samples (40). The increased ratio of crystalline region in the annealed starch granules, which was induced by the formation of new double helices, also increased the *T*_o_, *T*_p_, and *T*_c_ values (37). These were in agreement with the results obtained in Section 3.3. Moreover, the plastification of crystallinity and suppressed hydration were correlated to the decrease in *T*_c_-*T*_o_ (38). Compared to DAnn, the HAnn and PAnn had significantly increased gelatinization temperatures, which indicates a perfect crystalline structure resulting from greater reorganization of double helices (41). The rich H^+^ ions in PAW resulted in the formation of more hydrogen bonds, which hindered the plasticization of mung bean starch during ANN and led to the highest gelatinization values.

The Δ*H* represents the energy required for destroying the double helix order in the starch granule (42). Compared to NMBS, the higher Δ*H* values of annealed starches were due to the increase in ordered double helices formed by the organization of amylopectin, which required more energy to disrupt and was consistent with the XRD results (Figure 2). Other reasons for increasing Δ*H* values of annealed samples included amylose-amylose interaction, amylose-amylopectin interaction, and organization of crystalline regions (38). For HAnn and PAnn, more heterogeneous crystallites formed by alkaline environment and acidic substances with RONS (43), respectively, making Δ*H* values significantly higher than that of DAnn, which showed that ANN with HW or PAW further improved the thermal stability of mung bean starch.

### 3.9 Pasting properties of samples

The pasting parameters of native and annealed starches are summarized in Table 4. The viscosities of annealed starches markedly decreased in comparison to those of NMBS, while the PT significantly increased, and PAnn had the lowest and highest values. HAnn and PAnn had lower viscosities than those of DAnn, which was due to increased fragmentation of starch chains by alkaline and acid hydrolysis, respectively (44). These were in agreement with DSC analysis results, indicating that ANN restricted starch swelling, postponed the gelatinization, and improved its thermostability.

**Table 4:** Pasting properties of starch samples.

**Properties**	**Samples**			
	**NMBS**	**DAnn**	**HAnn**	**PAnn**
PV (cP)	4,155.0 ± 40.43^a^	3,982.5 ± 35.21^b^	2,783.0 ± 10.35^c^	1,984.0 ± 21.25^d^
BD (cP)	1,388.0 ± 36.50^a^	1,296.5 ± 6.32^b^	965.0 ± 13.26^c^	677.0 ± 41.0^d^
SB (cP)	2,076.5 ± 34.32^a^	1,895.0 ± 51.73^b^	1,129.0 ± 4.22^c^	854.0 ± 2.8^d^
FV (cP)	4,843.5 ± 28.71^a^	4,581.0 ± 45.57^b^	2,947.0 ± 12.53^c^	2,161.0 ± 43.8^d^
PT (°C)	74.0 ± 0.18^c^	78.2 ± 0.93^c^	81.4 ± 0.47^b^	85.3 ± 0.42^a^

Means of triplicate determination ± SD with different letters in the row within each property are significantly different (*p* < 0.05). “cP” is the rapid viscosity unit. PV: peak viscosity; BD: breakdown viscosity; FV: final viscosity; SB: setback viscosity; PT: pasting temperature.

Viscosity is critical for the application of starch in food processing. The changes in viscosity of starch under heating and shearing generally depend on starch granule friction, amylose leaching, and granule structural arrangement (45). The significant decrease in PV and BD of annealed starches was mainly related to the inhibited granule swelling based on the increased interactions among starch chains. The lower SP of annealed samples indicates hindered amylose leaching, which resulted in a decrease in viscosities. The enhanced crystallinity might be another factor for the decrease in both PV and BD. The FV represents the increase in starch viscosity after cooling, and SB indicates the retrogradation capacity of starch (46). Compared to NMBS, the annealed starches had significantly decreased FV and SB, which was attributed to the enhanced cross-linking among starch chains during ANN. Furthermore, the FV of HAnn and PAnn were markedly lower than that of DAnn. This might be due to a lack of time required for aligning the starch molecules with the flow direction during the pasting measurement. The substantial depolymerization of amylose also contributed to the reduction by interaction with the reactive species in PAW (26) and by the alkaline environment in HW (43). The decrease in SB indicated that ANN caused mung bean starch to adopt more stable conformations. Following ANN, the increased PT of annealed starches might be ascribed to several factors, including higher bond strength, increased crystallinity, more intermolecular cross-links, and reduced space among starch chains (27). Therefore, the above results revealed the reduction in the pseudoplastic nature and retrogradation tendency of mung bean starch after ANN, especially with PAW.

### 3.10 *In vitro* digestibility properties

**Figure 5:**
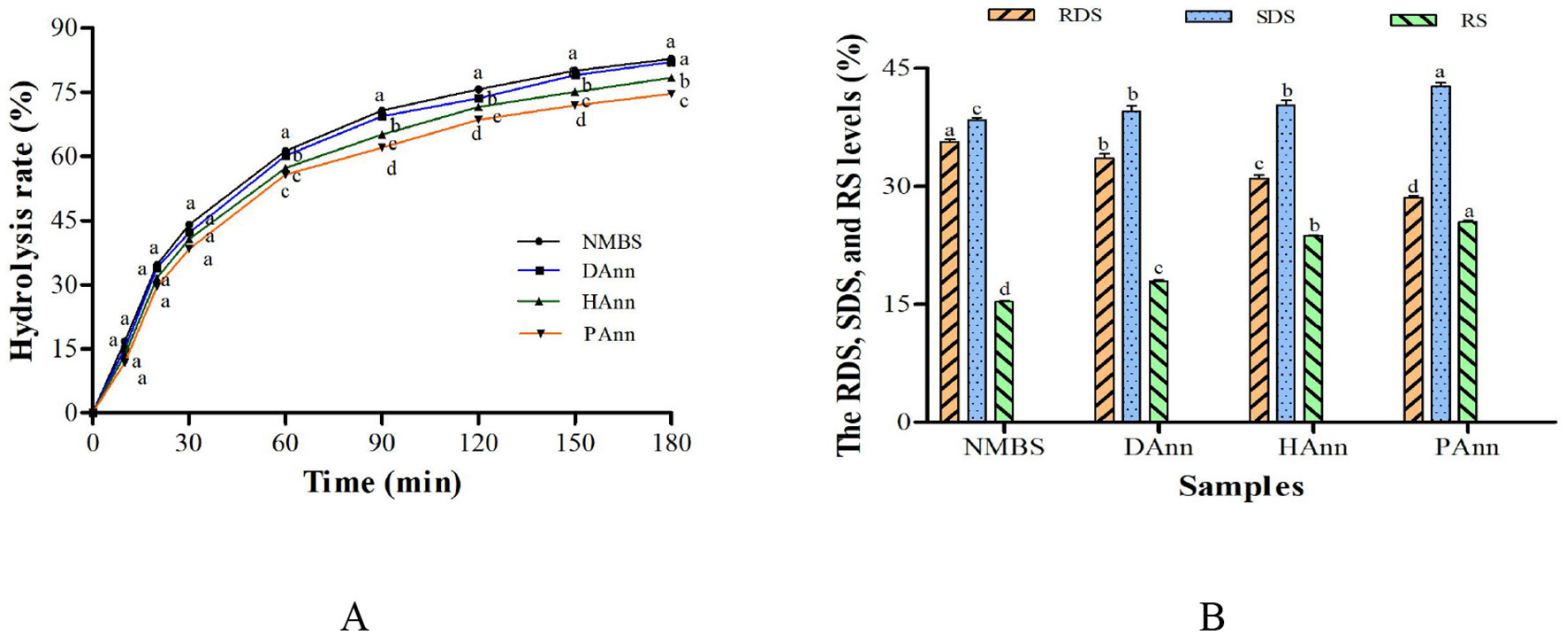
*In vitro* hydrolysis rate **(A)** and RDS, SDS, and RS levels **(B)** of native and annealed starches. Bars bearing different letters at the same time point **(A)** and different samples **(B)** within the same property are significantly different (*p* < 0.05).

The *in vitro* hydrolysis curve for all starches is shown in Figure 5A, and the levels of RDS, SDS, and RS are presented in Figure 5B. The hydrolysis rate of NMBS and annealed starches increased as the digestion time prolonged. Compared to the annealed samples, NMBS had the highest hydrolysis rate at each digestion time point. Starch is classified into RDS, SDS, and RS based on its digestion rate (47). After ANN, the RDS content markedly decreased, while the levels of SDS and RS increased; PAnn had the lowest RDS content (28.5%) and the highest SDS level (42.7%). In comparison to NMBS, the RS proportion in annealed starches significantly increased by 2.6% (DAnn), 8.4% (HAnn), and 10.1% (PAnn), respectively. These results indicate that ANN with HW and PAW had a much greater effect on starch digestibility than with DW.

The enzymatic susceptibility of annealed starches decreases based on the amylose to amylopectin ratio, crystalline structure, amylose-lipid complexation, and ANN conditions (28). The higher AMC and better crystalline perfection of annealed starches (shown in Table 2, Figure 2) resulted in a lower digestion rate than that of NMBS. Although the pores and fissures on the annealed sample surface facilitated digestive enzymes entering into granule interior to increase its hydrolysis rate, the results of *in vitro* digestibility revealed that the increment in AMC and RC could counteract the effect of morphological changes on starch hydrolysis. The increase in the content of SDS might also be due to increased crystalline perfection and molecular bonding in starch granules. The increased RS level was mainly ascribed to structural changes in granules, including starch chain interactions, higher RC, and molecular reorganization (48). These alterations significantly restricted the accessibility of starch molecules to enzymes, further decreasing the hydrolysis rate as well.

Meanwhile, the ANN with HW and PAW had a greater effect on starch than the ANN with distilled water. The reason might be based on the alkaline and acidic environment in HW and PAW, respectively, which produced lower-molecular-weight hydrolysates (such as oligosaccharides) that were resistant to the enzymatic hydrolysis. These results suggested that ANN was an effective way to decrease the hydrolysis rate of mung bean starch, and applying HW or PAW into ANN could improve more SDS and RS contents.

### 3.11 PCA of results

**Figure 6:**
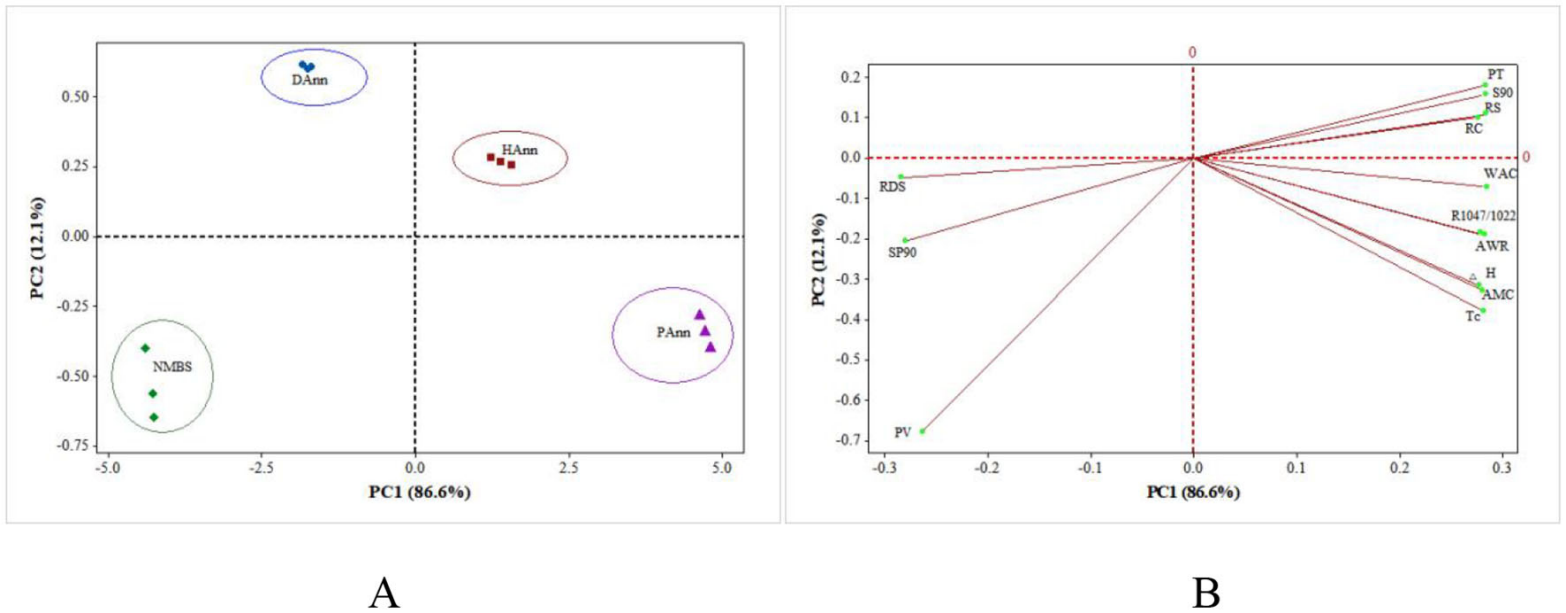
PCA biplots summarizing the relationships among samples and their structural, physicochemical, and digestibility properties. **(A)** Clusters of different samples on score plot; **(B)** PCA loading plot of different properties. PT: pasting temperature; S90: solubility at 90 °C; RS: resistant starch; RC: relative crystallinity; WAC: water absorption capacity; R_1047/1022_: the absorbance ratio of 1,047/1,022 cm^−1^ in FT-IR; AWR: alkaline water retention; Δ*H*: transition enthalpy; AMC: amylose content; *T*_c_: concluding temperature; RDS: rapidly digestible starch; SP90: swelling power at 90 °C; PV: peak viscosity.

The PCA of structural, physicochemical, and *in vitro* digestive results among NNBS, DAnn, HAnn, and PAnn is shown in Figure 6. The PC1 and PC2, respectively, represented 86.6% and 12.1% of the total variance, a total of 98.7%. The NMBS and DAnn were at the negative part of PC1, whereas HAnn and PAnn were at the positive part, as shown on the score plot (Figure 6A). Similarly, four samples were also located on two different sides of PC2. The distance between any two starches on the score plot was positively related to the degree of difference between them. Annealed starches gradually moved farther from NMBS, which revealed that ANN had various effects on structural, physicochemical, and *in vitro* digestive properties of NMBS. The relatively close distance between DAnn/HAnn and NMBS suggests that the effect of ANN depends on the type of water used, with PAnn being altered to the greatest extent. Furthermore, SP90, RDS, and PV stayed at the negative part of PC1 on the loading plot (Figure 6B) and were close to NMBS on the score plot, indicating that they were highly correlated. The PT, S90, RS, RC, WAC, R_1047/1022_, AWR, Δ*H*, AMC, and *T*_c_ were at the positive part of PC1 and positively related to HAnn and PAnn. These showed that ANN with HW or PAW had huge effects on the various structures, thermostability, physicochemical properties, and digestive functions of mung bean starch. Therefore, PCA verified a strong relationship among different samples, as well as showed some clusters.

## 4 Conclusion

The ANN with different types of water had important effects on multi-structures, physicochemical properties, and *in vitro* digestibility of mung bean starch. This modification significantly altered the morphological structure of NMBS, and the annealed starches had higher AMC, improved gelatinization characteristics and increased AWR, and lower SP at different temperatures. Although the “C”-type crystalline pattern of mung bean starch was not altered after ANN, the crystallinity significantly increased. Moreover, the annealed starches had higher pasting temperatures with better thermal stability than NMBS. All annealed starches revealed a decrease *in vitro* hydrolysis rate and less RDS content than NMBS, while having higher SDS and RS levels. Compared to distilled water, ANN with HW and PAW significantly enhanced these changes in NMBS to a much larger extent, with PAW having the greatest effect. This might be attributed to more interactions among starch molecules induced by hydrogen atoms and an acidic environment with RONS, respectively. The annealed starches with HW and PAW were more suitable than the sample with distilled water for producing soup, noodles, baked goods, and canned foods. The results of this study demonstrate that the joint use of ANN with HW or PAW not only provides an effective way for mung bean starch modification but also significantly expands the application of HW and PAW in other starch modification. Thus, further research is desired to uncover more possible underlying mechanisms of HW and PAW on starches.

## Data Availability

The original contributions presented in the study are included in the article/supplementary material, further inquiries can be directed to the corresponding author.

## References

[1] Ganesan K Xu B. A critical review on phytochemical profile and health promoting effects of mung bean (*Vigna radiata*). Food Sci Hum Well. (2018) 7:11–33. 10.1016/j.fshw.2017.11.002

[2] Tang D Dong Y Ren H Li L He C. A review of phytochemistry, metabolite changes, and medicinal uses of the common food mung bean and its sprouts (*Vigna radiata*). Chem Cent J. (2014) 8:4. 10.1186/1752-153X-8-4 24438453 PMC3899625

[3] Hou D Yousaf L Xue Y Hu J Wu J Hu X. Mung Bean (*Vigna radiata* L): bioactive polyphenols, polysaccharides, peptides, and health benefits. Nutrients. (2019) 11:1238. 10.3390/nu11061238 31159173 PMC6627095

[4] Shi Z Yao Y Zhu Y Ren G. Nutritional composition and antioxidant activity of twenty mung bean cultivars in China. Crop J. (2016) 4:398–406. 10.1016/j.cj.2016.06.011

[5] Li W Shu C Zhang P Shen Q. Properties of starch separated from ten mung bean varieties and seeds processing characteristics. Food Bioprocess Tech. (2011) 4:814–21. 10.1007/s11947-010-0421-6

[6] Ferreira CD Ziegler V da Silva Lindemann I Hoffmann JF Vanier NL de Oliveira M. Quality of black beans as a function of long-term storage and moldy development: chemical and functional properties of flour and isolated protein. Food Chem. (2018) 246:473–80. 10.1016/j.foodchem.2017.11.118 29291875

[7] Liang S Su C Saleh AS Wu H Zhang B Ge X. Repeated and continuous dry heat treatments induce changes in physicochemical and digestive properties of mung bean starch. J Food Process Pres. (2021) 45:e15281. 10.1111/jfpp.15281

[8] Duyen TTM Huong NTM Phi NTL Van Hung P. Physicochemical properties and *in vitro* digestibility of mung-bean starches varying amylose contents under citric acid and hydrothermal treatments. Int J Biol Macromol. (2020) 164:651–8. 10.1016/j.ijbiomac.2020.07.187 32702422

[9] Zhao K Zhang B Su C Gong B Zheng J Jiang H. Repeated heat-moisture treatment: a more effectiveway for structural and physicochemical modification of mung bean starch compared with continuous way. Food Bioprocess Tech. (2020) 13:452–61. 10.1007/s11947-020-02405-029196012

[10] Li W Zhang F Liu P Bai Y Gao L Shen Q. Effect of high hydrostatic pressure on physicochemical, thermal and morphological properties of mung bean (*Vigna radiata* L). Starch J Food Eng. (2011) 103:388–93. 10.1016/j.jfoodeng.2010.11.008

[11] Huong NTM Hoa PN Van Hung P. Effects of microwave treatments and retrogradation on molecular crystalline structure and *in vitro* digestibility of debranched mung-bean starches. Int J Biol Macromol. (2021) 190:904–10. 10.1016/j.ijbiomac.2021.09.032 34534585

[12] Liu H Wang L Shen M Guo X Lv M Wang M. Changes in physicochemical properties and in vitro digestibility of tartary buckwheat and sorghum starches induced by annealing. Starch-Stärke. (2016) 68:709–18. 10.1002/star.201500261

[13] Chung HJ Liu Q Hoover R. Impact of annealing and heat-moisture treatment on rapidly digestible, slowly digestible and resistant starch levels in native and gelatinized corn, pea and lentil starches. Carbohyd Polym. (2009) 75:436–47. 10.1016/j.carbpol.2008.08.006

[14] Okyere AY Rajendran S Annor GA. Cold plasma technologies: their effect on starch properties and industrial scale-up for starch modification. Curr Res Food Sci. (2022) 5:451–63. 10.1016/j.crfs.2022.02.007 35243357 PMC8866071

[15] Thirumdas R Kothakota A Annapure U Siliveru K Blundell R Gatt R. Plasma activated water (PAW): chemistry, physico-chemical properties, applications in food and agriculture. Trends Food Sci Tech. (2018) 77:21–31. 10.1016/j.tifs.2018.05.007

[16] Ohsawa I Ishikawa M Takahashi K Watanabe M Nishimaki K Yamagata K. Hydrogen acts as a therapeutic antioxidant by selectively reducing cytotoxic oxygen radicals. Nat Med. (2007) 13:688–94. 10.1038/nm1577 17486089

[17] Buchholz B Kaczorowski D Sugimoto R Yang R Wang Y Billiar T. Hydrogen inhalation ameliorates oxidative stress in transplantation induced intestinal graft injury. Am J Transplant. (2008) 8:2015–24. 10.1111/j.1600-6143.2008.02359.x 18727697

[18] Liu Z Fu Y Zhang J Shen Q. Comparison on physicochemical properties of mung bean flour and isolated starch under different level of high static pressure. Cereal Chem. (2021) 98:1203–14. 10.1002/cche.10472

[19] Liu H Lv M Peng Q Shan F Wang M. Physicochemical and textural properties of tartary buckwheat starch after heat–moisture treatment at different moisture levels. Starch-Stärke. (2015) 67:276–84. 10.1002/star.201400143

[20] Adebowalea KO Olu-Owolabi BI Olayinka OO Lawal OS. Effect of heat moisture treatment and annealing on physicochemical properties of red sorghum starch. Afr J Biotechnol. (2005) 4:928–33.

[21] Juliano BO Perez CM Blakeney AB Castillo T Kongseree N Laignelet B. International cooperative testing on the amylose content of milled rice. Starch-Stärke. (1981) 33:157–62. 10.1002/star.19810330504

[22] Liu H Wang LJ Cao R Fan H Wang M. *In vitro* digestibility and changes in physicochemical and structural properties of common buckwheat starch affected by high hydrostatic pressure. Carbohyd Polym. (2016) 144:1–8. 10.1016/j.carbpol.2016.02.028 27083786

[23] Adhikari B Adhikari M Ghimire B Park G Choi EH. Cold atmospheric plasma-activated water irrigation induces defense hormone and gene expression in tomato seedlings. Sci Rep. (2019) 9:16080. 10.1038/s41598-019-52646-z 31695109 PMC6834632

[24] Wu S Zhang Q Ma R Yu S Wang K Zhang J. Reactive radical-driven bacterial inactivation by hydrogen-peroxide-enhanced plasma-activated-water. Eur Phys J Special Topics. (2017) 226:2887–99. 10.1140/epjst/e2016-60330-y27627411

[25] Xu Y Tian Y Ma R Liu Q Zhang J. Effect of plasma activated water on the postharvest quality of button mushrooms, *Agaricus bisporus*. Food Chem. (2016) 197:436–44. 10.1016/j.foodchem.2015.10.144 26616972

[26] Aaliya B Sunooj KV Navaf M Akhila PP Sudheesh C Sabu S. Influence of plasma-activated water on the morphological, functional, and digestibility characteristics of hydrothermally modified non-conventional talipot starch. Food Hydrocolloid. (2022) 130:107709. 10.1016/j.foodhyd.2022.107709

[27] Yan Y Peng B Niu B Ji X He Y Shi M. Understanding the structure, thermal, pasting, and rheological properties of potato and pea starches affected by annealing using plasma-activated water. Front Nutr. (2022) 9:842662. 10.3389/fnut.2022.842662 35198591 PMC8859486

[28] Zavareze EdR Dias ARG. Impact of heat-moisture treatment and annealing in starches: a review. Carbohyd Polym. (2011) 83:317–28. 10.1016/j.carbpol.2010.08.064

[29] Zou J Xu M Wang R Li W. Structural and physicochemical properties of mung bean starch as affected by repeated and continuous annealing and their *in vitro* digestibility. Int J Food Prop. (2019) 22:898–910. 10.1080/10942912.2019.1611601

[30] Liu H Gao S Tian G Zhang S Liu S. Comparative study: how dry heating treatment and annealing influence the multi-structure, physicochemical properties and *in vitro* digestibility of black highland barley starch. Front Nutr. (2024) 11:1453424. 10.3389/fnut.2024.1453424 39149549 PMC11324538

[31] Lorenz K Kulp K. Heat-moisture treatment of starches ii: functional properties and baking potential. In: The Effect of Heat-Moisture Treatment on the Structure and Physicochemical Properties of Legume Starches (Thesis). Department of Biochemistry, Memonal University of Newfoundland Canada, St. John's, NL, Canda (1981).

[32] Falade KO Ayetigbo OE. Effects of annealing, acid hydrolysis and citric acid modifications on physical and functional properties of starches from four yam (*Dioscorea* spp) cultivars. Food Hydrocolloid. (2015) 43:529–39. 10.1016/j.foodhyd.2014.07.008

[33] Sathe S Salunkhe DK. Isolation, partial characterization and modification of the great northern bean (*Phaseolus vulgaris* L) starch. J Food Sci. (1981) 46:617–21. 10.1111/j.1365-2621.1981.tb04924.x

[34] Jayakody L Hoover R. Effect of annealing on the molecular structure and physicochemical properties of starches from different botanical origins–a review. Carbohyd Polym. (2008) 74:691–703. 10.1016/j.carbpol.2008.04.032

[35] Gao S Liu H Sun L Cao J Yang J Lu M. Rheological, thermal and in vitro digestibility properties on complex of plasma modified tartary buckwheat starches with quercetin. Food Hydrocolloid. (2021) 110:106209. 10.1016/j.foodhyd.2020.106209

[36] Rocha-Villarreal V Hoffmann JF Vanier NL Serna-Saldivar SO García-Lara S. Hydrothermal treatment of maize: changes in physical, chemical, and functional properties. Food Chem. (2018) 263:225–31. 10.1016/j.foodchem.2018.05.003 29784311

[37] Waduge R Hoover R Vasanthan T Gao J Li J. Effect of annealing on the structure and physicochemical properties of barley starches of varying amylose content. Food Res Int. (2006) 39:59–77. 10.1016/j.foodres.2005.05.008

[38] Tester R Debon S Sommerville M. Annealing of maize starch. Carbohyd Polym. (2000) 42:287–99. 10.1016/S0144-8617(99)00170-8

[39] Okyere AY Boakye PG Bertoft E Annor GA. Temperature of plasma-activated water and its effect on the thermal and chemical surface properties of cereal and tuber starches. Curr Res Food Sci. (2022) 5:1668–75. 10.1016/j.crfs.2022.09.020 36193040 PMC9526130

[40] Zhang B Wu C Li H Hu X Jin Z Tian Y. Long-term annealing of C-type kudzu starch: effect on crystalline type and other physicochemical properties. Starch-Stärke. (2015) 67:577–84. 10.1002/star.201500003

[41] Vamadevan V Bertoft E Soldatov DV Seetharaman K. Impact on molecular organization of amylopectin in starch granules upon annealing. Carbohyd Polym. (2013) 98:1045–55. 10.1016/j.carbpol.2013.07.006 23987446

[42] Cooke D Gidley MJ. Loss of crystalline and molecular order during starch gelatinisation: origin of the enthalpic transition. Carbohyd Res. (1992) 227:103–12. 10.1016/0008-6215(92)85063-6

[43] Xu Z Liu X Zhang C Ma M Gebre BA Mekonnen SA. Mild alkali treatment alters structure and properties of maize starch: the potential role of alkali in starch chemical modification. Int J Biol Macromol. (2024) 274:133238. 10.1016/j.ijbiomac.2024.133238 38897493

[44] Zambelli RA Galvão AMMT de Mendonça LG de Souza Leão MV Carneiro SV Lima ACS. Effect of different levels of acetic, citric and lactic acid in the cassava starch modification on physical, rheological, thermal and microstructural properties. Food Sci Technol Res. (2018) 24:747–54. 10.3136/fstr.24.74737595691

[45] Sasaki T Yasui T Matsuki J Satake T. Comparison of physical properties of wheat starch gels with different amylose content. Cereal Chem. (2002) 79:861–6. 10.1094/CCHEM.2002.79.6.861

[46] Balet S Guelpa A Fox G Manley M. Rapid Visco analyser (RVA) as a tool for measuring starch-related physiochemical properties in cereals: a review. Food Anal Method. (2019) 12:2344–60. 10.1007/s12161-019-01581-w

[47] Giri S Banerji A Lele S Ananthanarayan L. Effect of addition of enzymatically modified guar gum on glycemic index of selected Indian traditional foods (Idli, Chapatti). Bioactive Carbohyd Dietary Fibre. (2017) 11:1–8. 10.1016/j.bcdf.2017.05.002

[48] Kutoš T Golob T Kač M Plestenjak A. Dietary fibre content of dry and processed beans. Food Chem. (2003) 80:231–5. 10.1016/S0308-8146(02)00258-3

